# Supplementary figures and images for: A direct experimental test of Ohno’s hypothesis
Source: eLife. 2025 Apr 2;13:RP97216. doi: 10.7554/eLife.97216 (PMC11964449; doi:10.7554/eLife.97216)

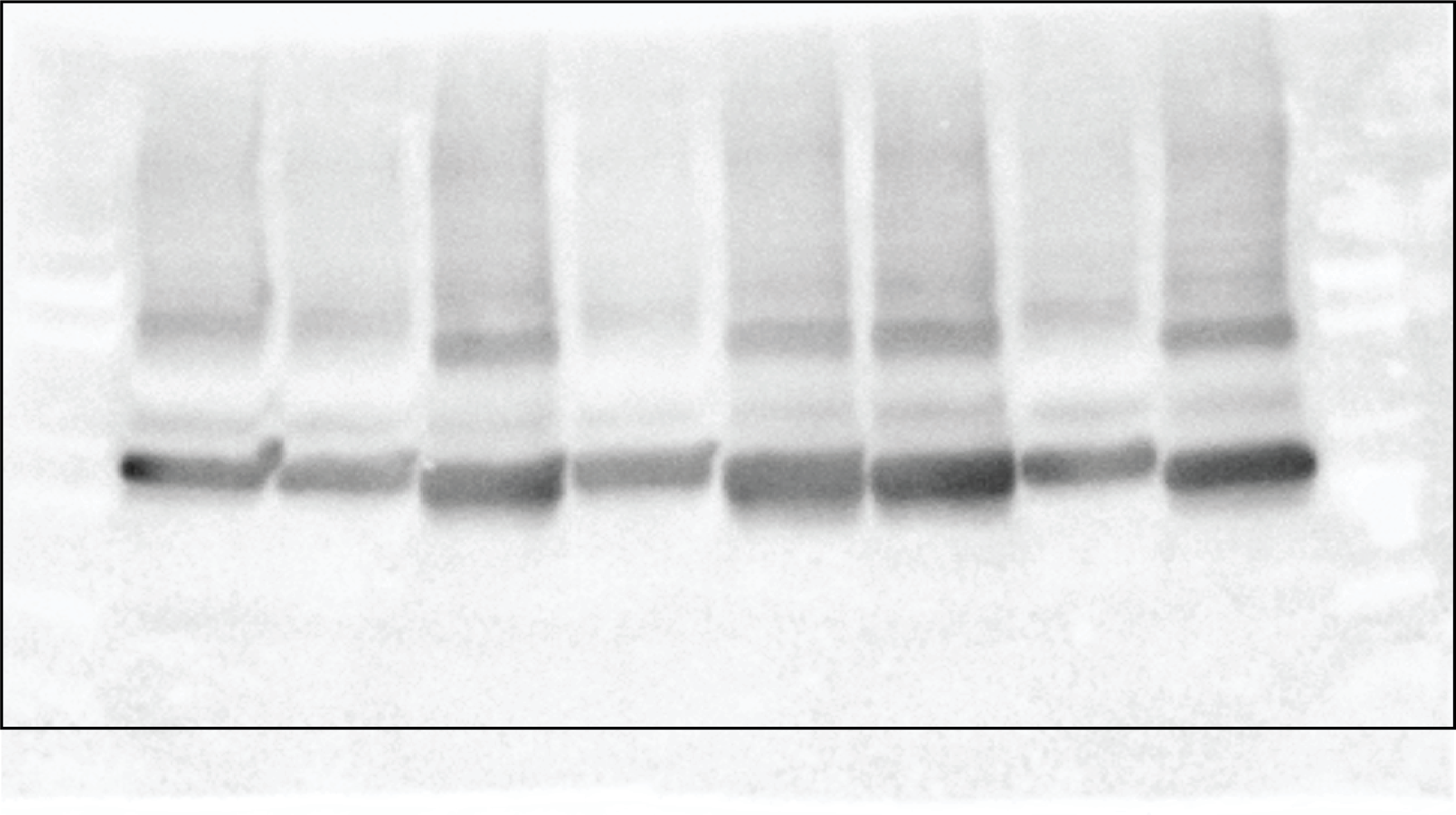

Supplement: Figure 6—figure supplement 4—source data 1. [file elife-97216-fig6-figsupp4-data1.zip › WB_uncropped_boundary.png]

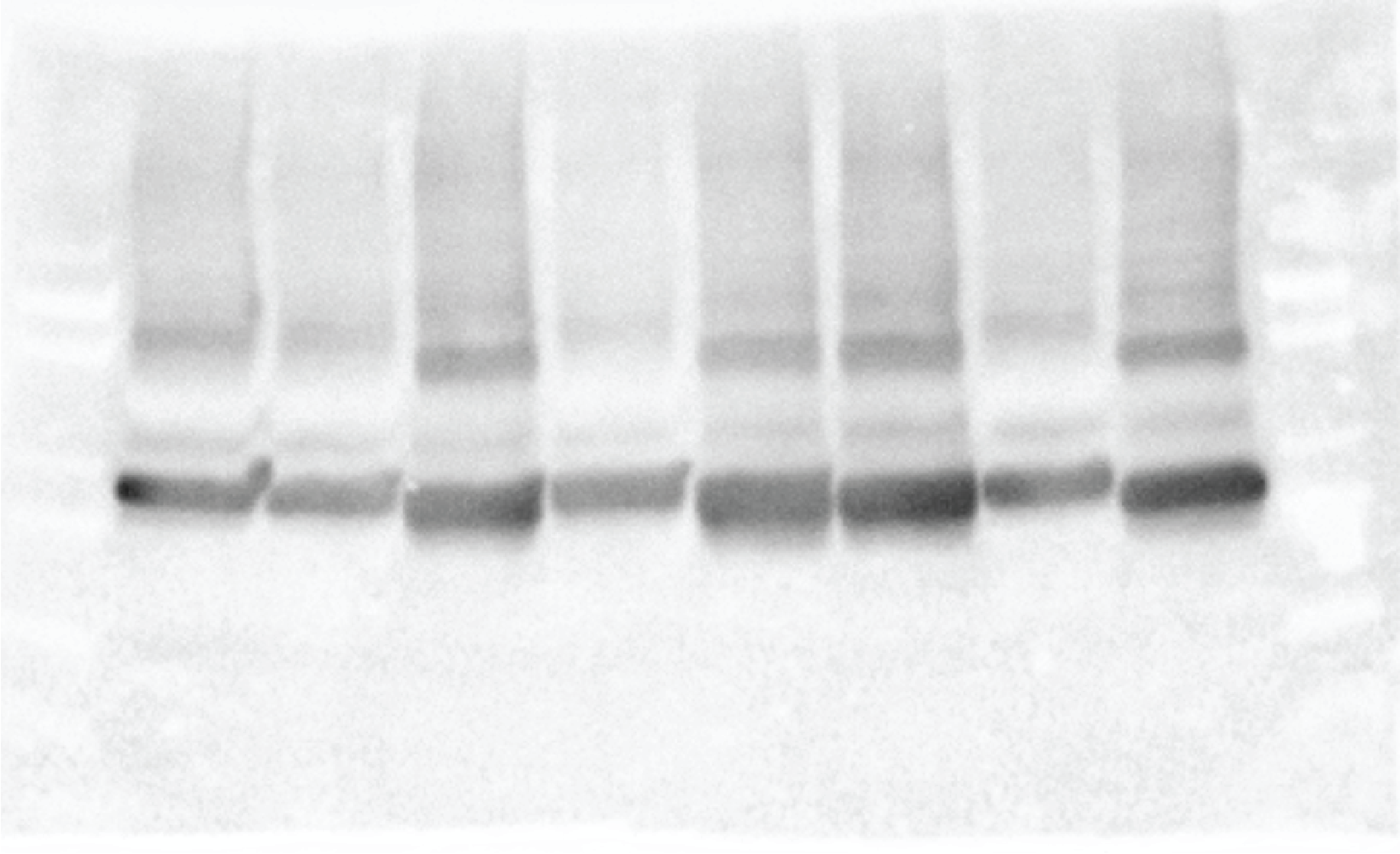

Supplement: Figure 6—figure supplement 4—source data 1. [file elife-97216-fig6-figsupp4-data1.zip › WB_uncropped.png]
